# Qualitative enquiry supporting trials: the 'quest' to integrate qualitative methods in clinical trials

**DOI:** 10.1186/1745-6215-14-S1-P97

**Published:** 2013-11-29

**Authors:** Clare Clement, Frances Rapport

**Affiliations:** 1 Swansea University, Swansea, UK

## 

Following mounting evidence for the value of qualitative research; trials are increasingly adopting mixed method approaches. Reviews suggest that whilst qualitative methods are being utilised within trials, methodological gaps, failure to integrate findings and variation in quality are ongoing issues. Clinical Trials Units (CTUs) have been established within the UK to "support, high quality, efficient, effective and sustainable clinical trials research". It is common practice for CTUs to provide statistical, quality assurance and health economics support but the formalisation of qualitative support is limited.

To address this within Wales, the QUalitative Enquiry Supporting Trials (QUEST) unit has been set up as the qualitative arm of the West Wales Organisation for Rigorous Trials in Health (WWORTH). Funded by the National Institute of Social Care and Health Research (NISCHR), QUEST aims to; provide qualitative support and resources to trial development and management groups and to ensure high quality, qualitative research becomes and integral part of clinical trials within Wales.

QUEST is collaborating with a number of trial groups. The Hughes Abdominal Repair Technique (HART) Trial; a surgical technique feasibility trial, provides an example of how QUEST has achieved the integration of qualitative methods within the overall trial design. Qualitative methods have been used to inform trial necessity, investigate patient and health professional views and the development of a condition specific Patient Reported Outcome Measure (PROMS).

The development of the qualitative arm of a clinical trials unit in Wales has led to increased implementation, appropriateness and quality of qualitative methods within trials.

